# Modulation of Renal Function in a Metabolic Syndrome Rat Model by Antioxidants in *Hibiscus sabdariffa* L.

**DOI:** 10.3390/molecules26072074

**Published:** 2021-04-04

**Authors:** Félix Leao Rodríguez-Fierros, Verónica Guarner-Lans, María Elena Soto, Linaloe Manzano-Pech, Eulises Díaz-Díaz, Elizabeth Soria-Castro, María Esther Rubio-Ruiz, Francisco Jiménez-Trejo, Israel Pérez-Torres

**Affiliations:** 1 Department of Cardiovascular Biomedicine, Instituto Nacional de Cardiología “Ignacio Chávez”, Juan Badiano 1, Sección XVI, Tlalpan, Mexico City 14080, Mexico; felixleao@hotmail.com (F.L.R.-F.); loe_mana@hotmail.com (L.M.-P.); elizabethsoria824@gmail.com (E.S.-C.); 2 Department of Physiology, Instituto Nacional de Cardiología “Ignacio Chávez”, Juan Badiano 1, Sección XVI, Tlalpan, Mexico City 14080, Mexico; gualanv@yahoo.com (V.G.-L.); esther_rubio_ruiz@yahoo.com (M.E.R.-R.); 3 Department of Immunology, Instituto Nacional de Cardiología “Ignacio Chávez”, Juan Badiano 1, Sección XVI, Tlalpan, Mexico City 14080, Mexico; mesoto50@hotmail.com; 4 Department of Reproductive Biology, Instituto Nacional de Ciencias Médicas y Nutrición Salvador Zubirán, Vasco de Quiroga 15, Sección XVI, Tlalpan, Mexico City 14000, Mexico; eulisesd@yahoo.com; 5 Department of Reproductive Biology, Instituto Nacional de Pediatría, Insurgentes Sur No. 3700-C, Coyoacán, Mexico City 04530, Mexico; trejofj@hotmail.com

**Keywords:** metabolic syndrome, *Hibiscus sabdariffa* L., oxidative stress, kidney, renal function

## Abstract

Metabolic syndrome (MS) is the association of three or more pathologies among which obesity, hypertension, insulin resistance, dyslipidemia, and diabetes are included. It causes oxidative stress (OS) and renal dysfunction. *Hibiscus sabdariffa* L. (HSL) is a source of natural antioxidants that may control the renal damage caused by the MS. The objective of this work was to evaluate the effect of a 2% HSL infusion on renal function in a MS rat model induced by the administration of 30% sucrose in drinking water. 24 male Wistar rats were divided into 3 groups: Control rats, MS rats and MS + HSL rats. MS rats had increased body weight, systolic blood pressure, triglycerides, insulin, HOMA index, and leptin (*p* ≤ 0.04). Renal function was impaired by an increase in perfusion pressure in the isolated and perfused kidney, albuminuria (*p* ≤ 0.03), and by a decrease in clearance of creatinine (*p* ≤ 0.04). The activity of some antioxidant enzymes including the superoxide dismutase isoforms, peroxidases, glutathione peroxidase, glutathione-S-transferase was decreased (*p* ≤ 0.05). Lipoperoxidation and carbonylation were increased (*p* ≤ 0.001). The nitrates/nitrites ratio, total antioxidant capacity, glutathione levels and vitamin C were decreased (*p* ≤ 0.03). The treatment with 2% HSL reversed these alterations. The results suggest that the treatment with 2% HSL infusion protects renal function through its natural antioxidants which favor an improved renal vascular response. The infusion contributes to the increase in the glomerular filtration rate, by promoting an increase in the enzymatic and non-enzymatic antioxidant systems leading to a decrease in OS and reestablishing the normal renal function.

## 1. Introduction

Metabolic syndrome (MS) is the association of three or more pathologies including obesity, hypertension, insulin resistance (IR), dyslipidemia and diabetes mellitus that occur simultaneously or sequentially in an individual. It causes alterations to organs and systems and it increases the risk of developing cardiovascular, kidney and liver diseases often leading to premature death [1]. The association between MS and kidney damage is related to obesity, IR, and oxidative stress (OS). In MS, the adipose tissue and the liver release pro-inflammatory cytokines and free fatty acids that affect the kidney causing glomerulosclerosis, nephrolithiasis, mesangial cell proliferation and loss of the morphological structure of the nephrons. These molecules also imbalance the response to vasoconstrictor and vasodilator agents in the renal afferent artery thus promoting alterations of the renal blood flow and glomerular ultra-filtration. These changes increase intraglomerular pressure, therefore allowing the passage of high molecular weight molecules such as albumin [2].

On another hand, reactive oxygen species (ROS) are molecules that occur naturally within biological systems and participate in various cellular and immunological activities that benefit the body [3]. However, an excessive increase in their level, caused by different pathologies such as those that comprise MS, leads to OS. This condition alters cellular metabolism through damage to proteins, lipids, enzymes, and DNA [4]. ROS are formed from molecular oxygen in the mitochondria, and through different enzymes such as xanthine oxidase, NADPH oxidase, cytochrome P450 and the inducible nitric oxide synthase (iNOS). The loss of renal function by ROS is due to endothelial dysfunction, damage to the structure of the cells in the glomeruli and renal tubules, inflammation, fibrosis and to genotoxic factors. ROS that participates in renal damage include O_2_^−^, H_2_O_2_, ONOO^−^ and OH^−^ [5].

However, the body possesses enzymatic and non-enzymatic antioxidant systems to eliminate the excess of free radicals. Some of the enzymes that form part of the enzymatic antioxidant system are the superoxide dismutase isoforms (SOD). These enzymes detoxify and protect the cell from the superoxide anion (O_2_^−^) anion by catalyzing the dismutation of two of these free radicals by a metallic cofactor that includes manganese, copper, or zinc. The final product of this reaction is the generation of H_2_O_2_ [6]. H_2_O_2_ is a molecule capable of diffusing through membranes; it has a long half-life and may act as a second messenger in different signaling pathways [7]. It initiates immediate effects such as changes in cell structure, cell proliferation, and recruitment of immune cells. It also damages lipidic membranes and causes necrosis, and apoptosis [8]. However, to detoxify this free radical, the enzymatic antioxidant system is provided with catalase (CAT), peroxidases and glutathione peroxidase (GPx). CAT is a very stable antioxidant enzyme which is not subject to denaturation and proteolysis due to its rigid structure. It has a high activity in the liver and erythrocytes and an average activity in the kidney and adipose tissue [9]. The glutathione peroxidase (GPx) isoform family consists of homologous enzymes that contain a selenocysteine. They are more effective than CAT in the removal of intracellular peroxides under many physiological conditions, and can reduce lipid hydrogen peroxides, and decrease lipid peroxidation (LPO) [10]. Another group of molecules that interact with H_2_O_2_ are peroxidases. They have an iron atom in the ferric form (Fe^3+^ or Fe^III^). Peroxidases play an important role in innate immunity and in many physiologically important processes such as apoptosis, inflammation and cell signaling. The activity of peroxidases is decreased in MS [11].

Another enzyme that participates in the detoxification process is glutathione-S-transferase (GST). This enzyme belongs to a superfamily of isoenzymes that include the (alpha [α], mu [μ], pi [π], omega [ω], theta [θ], delta [δ], zeta [ς], sigma [σ], kappa [κ] and the mitochondrial [m] forms. They are multifunctional and are found in the cytoplasm, mitochondria, endoplasmic reticulum, nucleus, cell membrane and in plasma. They function as protective enzymes for endogenous and exogenous cellular detoxification and they neutralize ROS [12]. This detoxification is carried out mainly through enzymatic conjugation with glutathione (GSH) to convert ROS into more soluble compounds that are more easily eliminated [13]. GSH is widely distributed in the cytoplasm and it reacts with ROS. It may also be oxidized by GPx and GST to form glutathione disulfide (GSSG) [14]. After participating in antioxidant functions, it is again reduced by the glutathione reductase (GR) [15].

Furthermore, plants are a source of natural antioxidants. They may function as alternative treatments to control the oxidative damage caused by MS in the kidney. In this sense, *Hibiscus sabdariffa* L. (HSL), also known as Jamaica flower, is a medicinal plant widely used in traditional medicine in Asia, Africa, and America. It contains polyphenols, anthocyanins, protocatechuic acid, epigallocatechins, resveratrol, and flavonoids, which protect against the different pathologies that comprise MS [16]. The effects of different fractions of the HSL infusion at the renal level have been reported in various studies. As an example, polyphenols obtained from HSL improve water exchange and reduce kidney damage in rats with diabetes induced by streptozotocin [17]. Another study showed that 2% HSL treatment decreased the infiltration of inflammatory cells, the level of NF-κB, and the expression of the genes of the enzymes involved in the production of prostaglandins in BALB/mice with kidney damage caused by LPS, [18]. Therefore, the aim of this work was to evaluate the effect of a 2% HSL infusion on renal function in a MS rat model induced by the administration of 30% sucrose in drinking water and evaluating the components of the antioxidant system in of kidney homogenate.

## 2. Results

MS rats showed significant increases in body weight (*p* ≤ 0.04), intra-abdominal fat, systolic blood pressure (SBP), triglycerides (TG), insulin, HOMA index, and leptin vs. the control rats (*p* = 0.001). The treatment with HSL in MS rats, significantly decreased the weight gain, SBP, TG levels, insulin, the marker of resistance to insulin (HOMA index) and leptin (*p* ≤ 0.04) in comparison with the MS rats. The glucose and cholesterol (CT) values did not show significant differences in any of the groups, (Table 1).

Table 2 describes the renal function markers. The MS rats showed deterioration of the renal function evidenced by a significant increase in serum creatinine (SCr), lower urine creatinine (UCr) and therefore decrease in creatinine clearance (CCr). They also had an increase in albuminuria (*p* ≤ 0.003) when compared with the control group. Furthermore, the MS rats showed an increase in water consumption when compared to the control and MS + HSL groups (*p* ≤ 0.003 and *p* ≤ 0.001 respectively). The HSL treatment in the MS rats resulted in a lower SCr, increase UCr and therefore an increase in CCr and a decreased in albuminuria (*p* ≤ 0.001).

**Figure 1:**
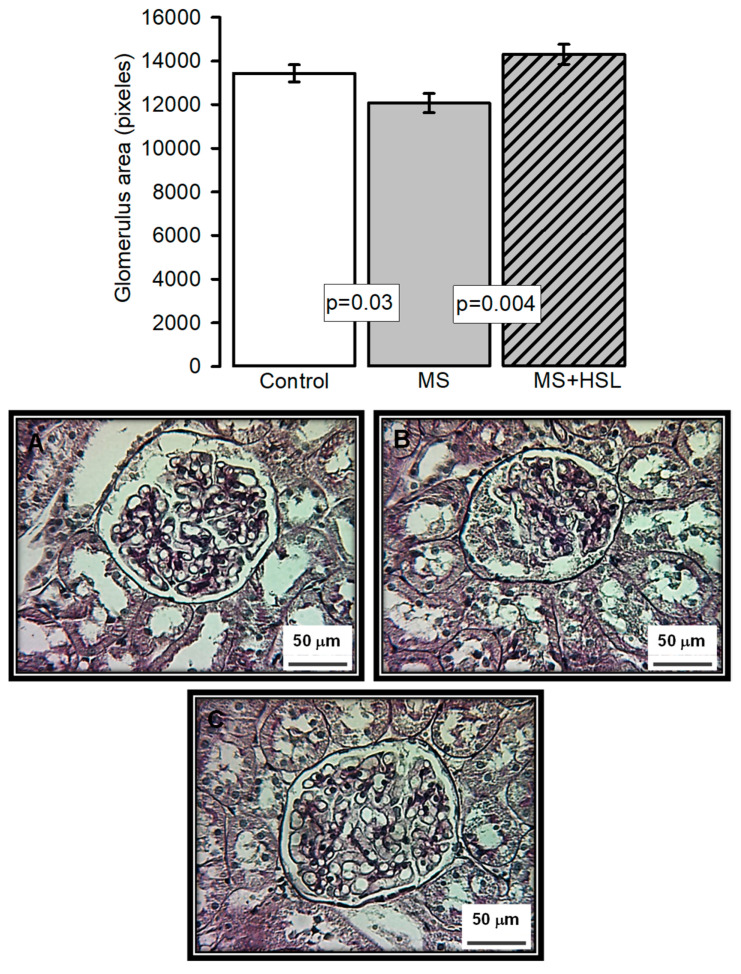
The top panel histogram that represents the densitometric analysis of the glomerular area in the experimental groups. Panel (A–C) Representative Photomicrographs of rat renal cortex. Images show the glomerulus from (**A**) control rats, (**B**) MS rats, and (**C**) MS + HSL. No abnormalities were observed under light microscopy in control and MS + HSL groups, where glomerular spaces and loops with their fine and delicate membrane are preserved. However, in the kidney of the MS rats showed retracted glomerular tuft, fibrosis, and an increased urinary space with detritus. (Periodic acid-Schiff stains, 400×).

The top panel in Figure 1 shows the average area size of the glomerular tang in each of the experimental groups. The densitometrical analysis showed a significant decrease in the size of the glomerulus in the MS rats when compared to the control and the MS + HSL groups (*p* = 0.03, *p* = 0.004 respectively). Figure 1A–C show representative histological sections of a glomerulus in the control, MS, and MS + HSL groups, respectively. The glomerular spaces and loops with their fine and delicate membrane are preserved in the control and MS + HSL groups in comparison with those from MS rats which show retracted glomerular tuft, fibrosis, and an increased urinary space with detritus.

**Figure 2:**
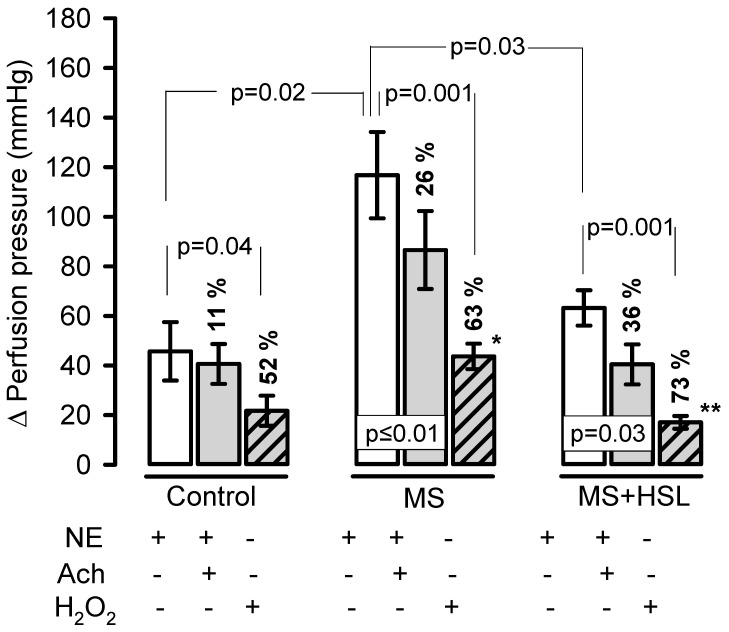
Changes the ∆ of the PP in the isolated kidney. The control, MS, and MS + HSL rats were perfused with NE 20 μM, in presence of (Ach 20 μM, and finally in presence of 1.7 μM of H_2_O_2_. Data are expressed as changes the delta (Δ) of the PP in mmHg. After each perfusion bolus, the kidneys were washed for a period of 20 min with Krebs solution, to allow them to return to the basal PP (75–90 mmHg). Statistically significant at * *p* = 0.01 control vs. MS and ** *p* = 0.001 MS + HSL vs. MS. Abbreviations: Ach = acetylcholine, NE = norepinephrine, MS = metabolic syndrome, MS + HSL = metabolic syndrome plus *Hibiscus sabdariffa* L., PP = pressure perfusion. Data are expressed in means ± SE, (*n* = 8, rats in each group). Statistical significance was determined by one-way ANOVA test, followed by Tukey’s post hoc test.

Figure 2 shows the changes in the Δ of PP, which increased significantly (*p* = 0.02) when NE was perfused into the kidney of MS rats in comparison with control rats (116.8 ± 49.1 mmHg vs. 45.7 ± 33.3 mmHg). The HSL treatment in MS rats resulted in a significant decrease (*p* = 0.03) in the Δ of PP when perfused with NE when compared to MS rats (63.2 ± 20.2 mmHg vs. 116.8 ± 49.1 mmHg). When NE was perfused in the presence of Ach, the Δ of PP decreased by 11% without significant difference in the control rats. The Δ of PP decreased in the kidney of the MS rats by 26% when compared to that present only with NE in the same group, with a significant difference (*p* = 0.01). However, when comparing the Δ of PP between the control and MS groups, a significant increase was observed (40.6 ± 22.8 mmHg vs 86.6 ± 44.4 mmHg, *p* = 0.01). In the MS + HSL rats, a decrease of 36% in the Δ of PP compared to NE in the same group (*p* = 0.03) was observed when adding NE in the presence of Ach. In addition, it showed a significant decrease in the Δ of PP compared to MS rats (40.4 ± 22.8 mmHg vs. 86.6 ± 44.5 mmHg, *p* = 0.01). However, when the NE was perfused in the presence of H_2_O_2_, a decrease in the Δ of PP of the 52% was observed in the control group when compared with NE from the same group with a significant difference (*p* = 0.04). However, in the MS group there was a significant decrease in the Δ of PP of up to 63% when compared to the vasoconstriction that was present with NE only, in the same group (*p* = 0.001). When the Δ of PP between the MS rats and the control rats was analyzed, a significant increase (43.7 ± 14.6 mmHg vs. 21.7 ± 17.2 mmHg, *p* = 0.01) was observed. In the MS + HSL group, there was a 73% decrease in the Δ of PP compared with that of the same group (*p* = 0.001), but when compared with the MS rats, a significant decrease in the Δ of PP (17.1 ± 7.2 mmHg vs. 43.7 ± 14.6 mmHg, *p* = 0.001) was found.

### Antioxidant Enzymes

**Figure 3:**
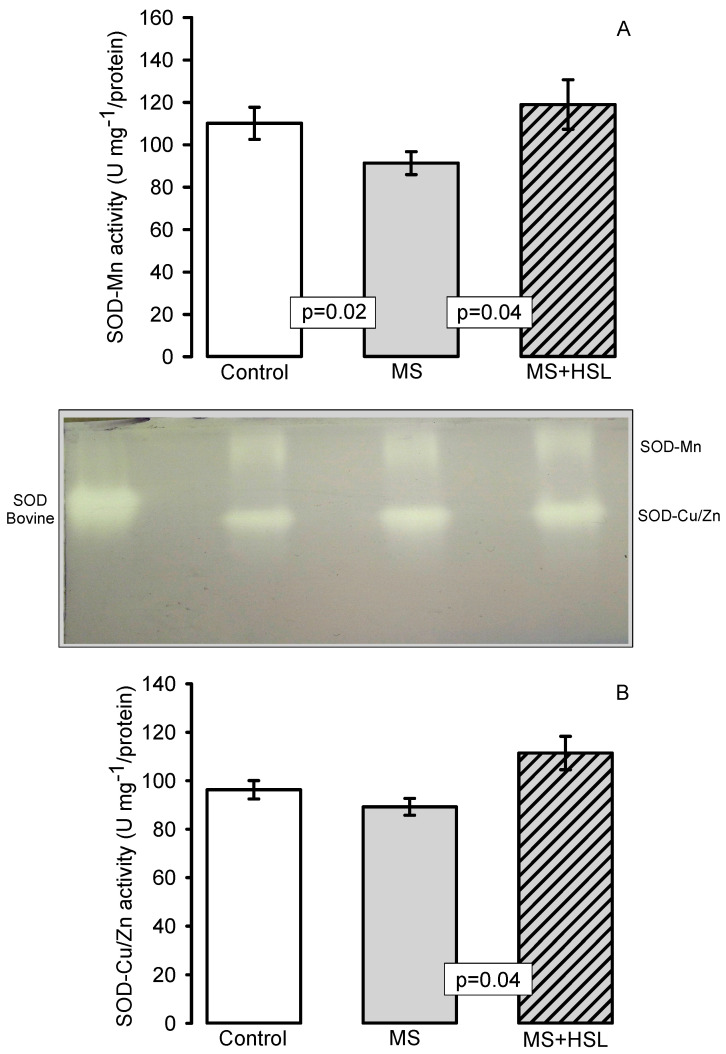
Histogram representing the densitophotometric analysis of the activity of the superoxide dismutase (SOD) isoforms in the kidney homogenates of the experimental rats. A representative native gel of the activities of the SOD-Mn, and SOD-Cu/Zn electrophoresis is shown between the graphs A and B is shown. (**A**) Mn isoform, and (**B**) Cu/Zn isoform. Abbreviations: MS = metabolic syndrome, MS + HSL = metabolic syndrome plus *Hibiscus sabdariffa* L., Mn = Manganese, Cu/Zn = Copper/Zinc Data are expressed in means ± SE, (*n* = 8, rats in each group). Statistical significance was determined.

Figure 3A,B shows the activity of the SOD isoforms. Panel A shows the SOD-Mn activity in the kidney homogenate of the experimental groups. In rats with MS, a decrease in SOD-Mn activity was observed when compared to the control and MS + HSL groups (*p* = 0.02 and *p* = 0.04, respectively). On the other hand, the SOD-Cu/Zn activity only showed significant changes between the MS rats and the rats of the MS + HSL group (*p* = 0.04).

**Figure 4:**
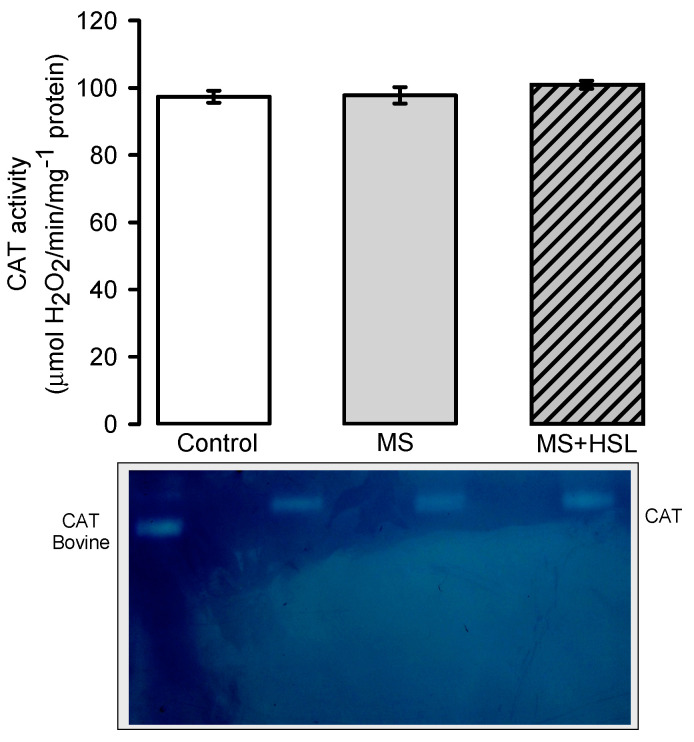
Densitophotometric analysis of the activity of catalase in the kidney homogenate. Below the histogram, a native representative gel of the CAT activity is included. Abbreviations: CAT = catalase, MS = metabolic syndrome, MS + HSL = metabolic syndrome plus *Hibiscus sabdariffa* L., Data are expressed in means ± SE, (*n* = 8, rats in each group).

Figure 4 shows the CAT activity, where no significant differences in any of the experimental groups were found.

**Figure 5:**
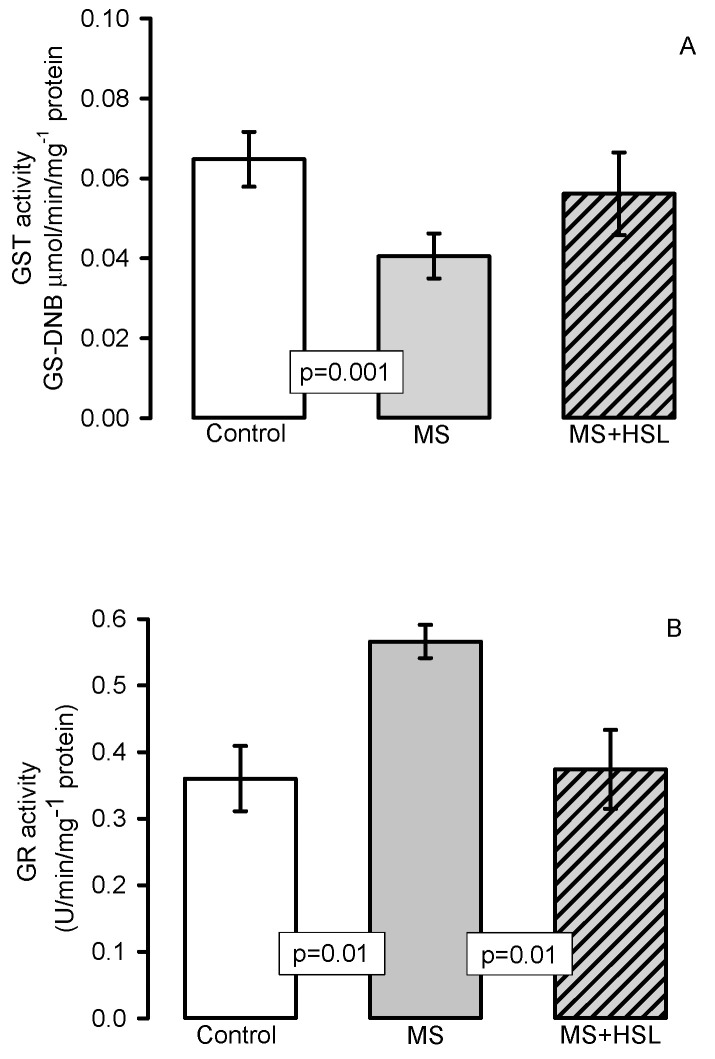
Effect of the HSL treatment on glutathione-S-transferase activity (panel **A**) and glutathione reductase (GR) activity (panel **B**) in kidney homogenate. Abbreviations: MS = metabolic syndrome, MS + HSL = metabolic syndrome plus *Hibiscus sabdariffa* L., GST = glutathione-S-transferase, GR = glutathione reductase. Data are expressed in means ± SE, (*n* = 8, rats in each group). Statistical significance was determined by one-way ANOVA test, followed by Tukey’s post hoc test.

Figure 5A shows the GST activity which was significantly lower in the MS rats compared to that found in the control rats (*p* = 0.001). However, between the MS + HSL vs. MS only a tendency to an increase was observed without reaching a statistically significant difference. Figure 5B shows the GR activity, in which there was a significant decrease in the MS rats when compared to the control and MS + HSL rats (*p* = 0.01).

**Figure 6:**
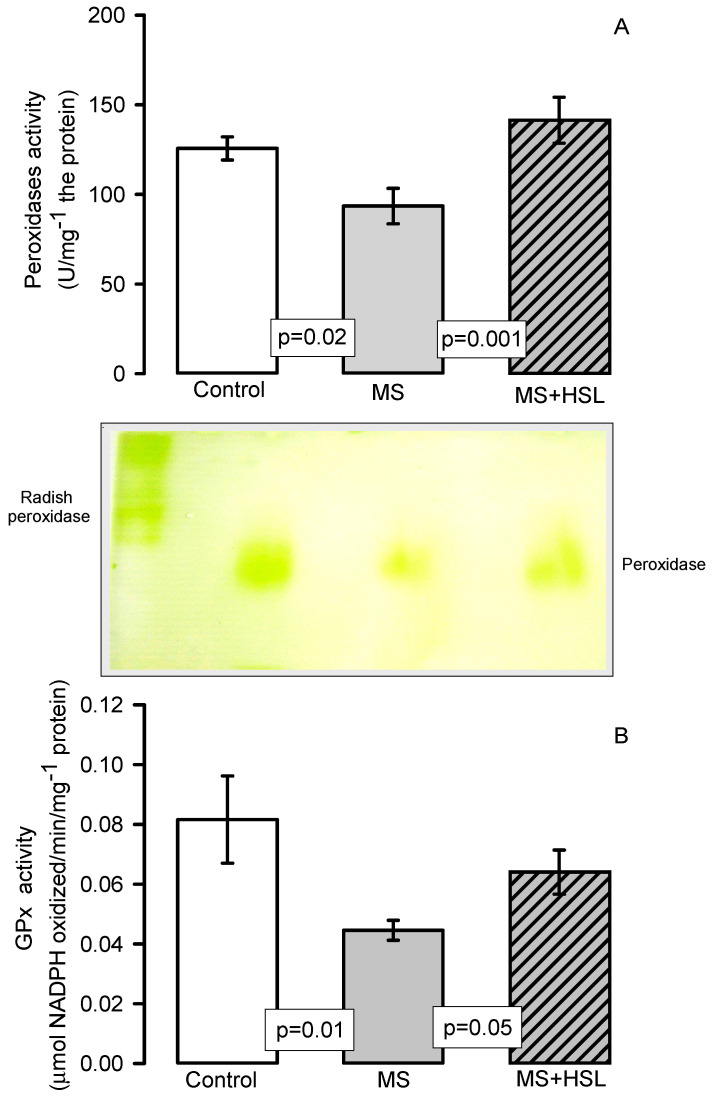
Peroxidase activity (**A**) and GPx activity (**B**). Panel (**A**) densitophotometric analysis of the activities of peroxidases, a representative native gel is shown below the histogram. Where peroxidases are present, the gel remains transparent and the 3, 3, 5, 5-tetramethylbenzidine is oxidized, showing a green coloration. Abbreviations: MS = metabolic syndrome, MS + HSL = metabolic syndrome plus *Hibiscus sabdariffa* L., GPx = glutathione peroxidase. Data are expressed in means ± SE, (*n* = 8, rats in each group). Statistical significance was determined by one-way ANOVA test, followed by Tukey’s post hoc test.

Figure 6A,B show the activity of peroxidases and GPx, respectively (Panel (A) by native gels and panel (B) by spectrophotometry). The peroxidase and GPx activities were determined by both techniques and showed a significant decrease in the MS rats in comparison with those found in the control and MS + HSL groups (*p* = 0.02, *p* = 0.001 and *p* = 0.01, *p* = 0.05 respectively).

The OS indicators in the kidney homogenate are shown in Table 3. There was a significant increase in LPO and in the carbonylation levels (*p* ≤ 0.001) and there were decreases in the NO_3_^−^/NO_2_^−^ ratio, GSH, TAC and Vit C levels (*p* ≤ 0.03 and *p* = 0.05 respectively) in the MS group in compared to the control group. The HSL treatment resulted in a decrease in LPO (*p* = 0.01) but an increase in NO_3_^−^/NO_2_^−^ ratio, GSH and Vit C levels (*p* = 0.001, *p* = 0.01 and *p* = 0.05 respectively) in comparison with the MS group.

## 3. Discussion

MS claims the lives of thousands of people every year around the world and therefore the search for new alternative and accessible treatments for this disease is of the greatest interest. OS plays an important role in the pathophysiology of MS, resulting in cellular damage and in hemodynamic alterations which affect the function of the kidney in the long term [2]. Several studies have focused on the use of traditional medicine to counteract the damage caused by ROS. In particular, the infusion with HSL has shown to decrease the OS in the liver and heart in MS rats [19]. Therefore, the aim of this work was to evaluate the effect of a 2% HSL infusion on renal function in a MS rat model. MS was induced by the administration of 30% sucrose in drinking water. We also determined the level of some components of the antioxidant system in the kidney homogenate.

### 3.1. Physiological Characteristics of the MS Model

The MS rat model courses with high systolic blood pressure (SBP), hypertriglyceridemia, obesity (intra-abdominal deposits of fat), hyperinsulinemia, and insulin resistance (IR) [19]. However, the HSL infusion decreased body weight, intra-abdominal fat, TG, insulin, HOMA index (IR marker), and leptin in the MS rats. This effect is attributed to the polyphenols, and flavonoids present in HSL. These antioxidant molecules have hypolipidemic effects inhibiting the accumulation of fat by reducing oxysteroles through the metabolism of bile acids. These components of HSL can block the lipid accumulation in the liver, adipose tissue and they can increase the palmitic acid excretion in the feces [20]. They increase the β-oxidation of fatty acids by inhibiting the fatty acid synthase, 3-hydroxy-3-methylglutaryl coenzyme A reductase, acyl-coenzyme A, and cholesterol acyltransferase, thus improving insulin sensitivity and reducing the TG levels [16]. Furthermore, the loss of intra-abdominal fat and the decrease in TG levels caused by the HSL infusion can be due in part, to the decrease in lipogenesis due to +/− hydroxycitrate (+/− HCA) that is transformed into −/− HCA. HCA inhibits the extra-mitochondrial enzyme ATP-citrate-lyase which catalyzes the breakdown of citrate to obtain oxaloacetate and acetyl-CoA. This is an important step for the synthesis of fat. However, these components can also inhibit the formation of malonyl-CoA that stimulates the activity of carnitine transferase leading to decreases in lipogenesis and increases the lipids oxidation and/or glycogenesis [21].

Leptin is an adipocytokine involved in energy expenditure, inflammation, lipid metabolism and glucose metabolism. Hyperlipidemia is positively associated to IR and to an increase in the intra-abdominal fat accumulation [22]. The treatment with the HSL infusion decreased leptin levels, suggesting that it may influence the synthesis leptin in adipose tissue [19]. A recent study showed that the administration of an HSL extract decreased the leptin mRNA levels in intra-abdominal adipocytes. Another study showed that the administration of the HSL in MS patients, reduced leptin levels [23].

In addition, IR, and hyperinsulinemia lead to structural abnormalities in the kidney, such as increased tubulointerstitial damage, fibrosis, and mesangial proliferation. These changes modify renal hemodynamics [24]. Our results show that the treatment with the HSL infusion decreased the insulin levels and the HOMA index in MS rats. This effect may be due to the presence of cyanidin-3-glucoside which can over express the GLUT4 transporter and may increase the signaling of insulin in the cells [25].

Furthermore, the MS rats showed hypertension and the 2 % HSL infusion treatment decreased SBP. The antihypertensive effects of HSL may be due to anthocyanins (delphinidin-3-O-sambubiosides and cyanidin-3-O-sambubioside) through different mechanisms. The first is the diuretic effect of these molecules that resembles that of aldosterone by increasing the elimination of water and sodium. The second mechanism could be by the inhibition of angiotensin converting enzyme 2 (ACE2) [26]. In addition to the antihypertensive effect of anthocyanins and polyphenols, these molecules can also induce the phosphorylation of eNOS in endothelial cells which contributes to decrease blood pressure [27]. Also, the administration of 2% HSL infusion in MS rats increased the secondary metabolites of nitric oxide (NO), the NO_3_^−^/NO_2_^−^ ratio. This suggests that these metabolites may be derived from the eNOS pathway and may contribute to the improvement of the endothelial function and the decrease in SBP. In this sense, the polyphenols present in HSL can activate the PI3K pathway in the endothelium and regulate NO through the phosphorylation of eNOS and the suppression of the mobilization of Ca^2+^, thus increasing vasorelaxation [28]. However, the protocatechuic acid is a potential inhibitor of the iNOS pathway [29].

### 3.2. Effect of a 2% HSL Infusion on the Kidney Function in MS

The MS rats showed deterioration of the renal function evidenced by an elevation of the levels of SCr, by albuminuria, by lowering CCr and by renal histological changes. Retracted glomerular tuft, fibrosis, and the increased urinary space with detritus were present. There are alterations in the renin angiotensin aldosterone system in MS, which are one of the main causes of the deterioration of the renal function. Changes in this system result in an increase in the intraglomerular pressure and filtration rate. These changes lead to an increase in SCr, albuminuria and a decrease in CCr [30]. However, The HSL infusion treatment improved renal function by lowering the concentration of SCr, and albuminuria, and by increasing CCr. The epithelial mesenchymal transition of human diabetic tubular cells promotes the generation of fibroblasts and favors fibrosis via up-regulation of AT−1 and the downstream TGF-β1 pathway. The polyphenols present in HSL attenuate the renal epithelial mesenchymal transition [31]. Administration of HSL in mice with renal damage caused by doxorubicin or adenine had decreased proteinuria, SCr and increased the CCr [32]. This HSL benefic effect has been associated to anthocyanins. These molecules inhibit the production of angiotensin II, and lead to an increase in the glomerular filtration rate [33]. HSL also contains quercetin and eugenol which are potent vasodilators that increase renal flow through increased levels of NO, produced via the eNOS pathway. In obese and diabetic rats with nephropathy, HSL administration decreases albuminuria and increases the CCr. These beneficial changes are associated to the preservation of the structural anatomy of the glomerulus [34]. Our results show that the treatment with HSL decreased the histopathological changes and improved renal function in the kidney of the MS rats. In addition, the administration of HSL in drinking water to mice with lipopolysaccharide-induced kidney inflammation, reduced histological changes such as tubular inflammation [18]. The administration of 5 and 10% the HSL extract in rats with adenine decreased inflammation and renal and interstitial tubule fibrosis [35].

### 3.3. Renal Vascular Resistance

The results in the isolated and perfused kidney showed that NE perfusion in MS rats was increased and there was an elevation in the Δ of PP. This could be due to the histopathological changes, to the loss of the renal function and to an increase in the α-adrenergic receptors. An elevation in these receptors leads to the alteration of the vascular reactivity in the afferent artery, causing an increase in renal vascular resistance [36]. NE acts by inhibiting the antihypertensive mechanisms including the release of renin, the elevation of natriuretic pressure, and the decrease in the release of the renal medullary depressant hormone [37]. However, HSL treatment in MS rats showed that the Δ of PP was lower when NE was perfused. This suggests that the HSL infusion decreases vascular resistance, probably by inhibiting the sensitivity of α-adrenergic receptors. In this sense, a decrease in the vasoconstrictive response in the presence of NE is present in isolated aortas from diabetic rats treated with an HSL extract [26]. Furthermore, an infusion of HSL showed a decrease the negative inotropic effect in isolated hearts of rats, probably through α-adrenergic receptors, and this was associated with a decrease in SBP.

Besides, the perfusion with NE+Ach decreased the percentage of the Δ of PP in the kidney of the rats treated with HSL in a 36% when compared to the MS rats in which it was of 26%. These results suggest the presence of endothelial damage in the renal vasculature in MS rats. They also suggest the beneficial effect of the HSL infusion on renal vascular reactivity by decreasing the Δ of PP. This is reflected in a decrease in renal vascular resistance. Previous studies demonstrated that this MS model courses with endothelial dysfunction, hyper contractility to NE and loss of Ach- dependent vasorelaxation in the aorta but that HSL treatment improved this condition [19]. In addition, these results can also be associated with the increase in the glomerular filtration rate and the level of CCr in the MS + HSL group. These beneficial effects of the HSL infusion can also be caused by the mediation of vasorelaxing mechanisms, by the inhibition of the ECA2, by a decrease of Ca^2+^ levels in vascular smooth muscle, by increases in NO via eNOS pathway and by a decrease in flux due to cholinergic and/or histaminergic factors [16,17].

The NE+H_2_O_2_ perfusion showed a decrease in the Δ of PP of 63% in MS rats; however, in the rats treated with the HSL infusion the decrease was of 73% (*p* = 0.001). These results suggest that MS rats’ course with OS, as has already been reported [38] and that the HSL infusion decreases this oxidant state. In this sense, H_2_O_2_ can have a dual effect by modulating vascular reactivity, and by a vasodilator effect in the sub mucosal arterioles [39]. Therefore, the effect depends on the vascular bed in which it is acting [40] and upon the physiological and pathological conditions [41]. The effect also depends on the concentration; at 10–100 nM, it has beneficial effects on the vascular reactivity; however, at high concentrations of 1 µM it induces vasoconstriction, inflammation, and cell death [42]. A study reported that low concentration of H_2_O_2_ activate 4-aminopyridine-sensitive potassium channels that lead to the closure of voltage-dependent Ca^2+^ channels. This causes hyperpolarization, which then leads to relaxation of arterial smooth muscle. However, increased OS impairs the capacity of H_2_O_2_ to exert its vasorelaxing effect [40]. H_2_O_2_ infused into the renal artery causes massive transient proteinuria [38]. A high production of H_2_O_2_ which acts as a potent renal vasoconstrictor [43], leads to hypertension in diabetic mice and rats [44]. This suggests that there is an excessive production of H_2_O_2_ in MS that contributes to renal damage. The excessive production of H_2_O_2_ may explain the differences in the response of the ∆ of PP. It may also be involved in the generation of the histopatological changes, glomerular hemodynamics, proteinuria, and hypertension which are present in the kidney of the MS rats.

### 3.4. Oxidant/Antioxidant Effect of the HSL Infusion

Our results show that there is a decrease in the activity of the enzymes SOD-Mn, peroxidases and GPx in the kidney of MS rats. These enzymes are associated with antioxidant defenses [45]. SOD-Cu/Zn did not show a significant difference, and this suggests a possible damage to the mitochondria in the kidney cells. This antioxidant enzyme (SOD-Mn) is located in the mitochondrial matrix and participates in the dismutation of the O_2_^−^ to H_2_O_2_ [46]. A decrease in the SOD activity is associated with MS regardless of cellular aging, OS markers and dietary risk factors. It even acts as a predictor of this syndrome [47]. Inhibition of SOD increases vascular OS and alters the endothelium-dependent vasoreactivity [48]. In mice lacking an isoform of SOD such as the extracellular isoform, there are increased amounts of ROS and alterations in vascular reactivity [49]. Down-regulation of SOD-Mn in MS rats and the subsequent excessive release of O_2_^−^ and H_2_O_2_ contribute to the inflammatory, proliferative, and fibrotic renal injury. However, the treatment with the HSL infusion increased the activity the two SOD isoforms. The polyphenols present in the HSL may neutralize ROS by donating an electron or hydrogen atom. They act as chelators and exert co-antioxidant activity with essential vitamins. They inhibit the xanthine oxidase and can also up-regulate SOD, GPx and peroxidase isoforms through the regulation of the Nrf2/Keap1 pathway. Furthermore, flavonoids are another type of molecule presents in HSL that could increase the activity of these enzymes [50]. The results suggest that the 2% HSL treatment increases the activity of SOD isoforms in the kidney, thus contributing to reduce OS in MS rats and to improve the kidney function. A recent study showed that the beneficial effect of the polyphenols present in HSL can be due to a lower generation of H_2_O_2_ in the arterioles. H_2_O_2_ interacts exogenously with endothelial cell, and penetrates through aquaporins inhibiting phosphatases, NADPH oxidases and activating Nrf2 [41]. However, the increase in the activity of the SOD isoforms could lead to an increase in the endogenous H_2_O_2_ in the kidney.

The activity of the SOD isoforms is related to the activity of other antioxidant enzymes such as CAT, GPx and peroxidases [51]. These enzymes are responsible for the elimination of the H_2_O_2_ which results from the activity of the SOD isoforms. The activity of these enzymes decreases OS in MS. GPx are the most efficient for the detoxification and removal of intracellular H_2_O_2_ and organic peroxides. CAT depends on the gradient and concentration of H_2_O_2_ [52]. Our results show that the activity of CAT did not show significant differences in the kidney of MS rats and therefore, the HSL treatment did not modify its activity. GPx do not depend on the concentration gradient but on the presence of NADPH and GSH for the conversion of H_2_O_2_ to H_2_O [19]. Therefore, they have a better antioxidant capacity to detoxify the H_2_O_2_ than CAT [53]. The GPx are predominantly localized in the renal tubules and an increase in their activity contributes to decrease the concentration of ROS and to improve renal blood flow [54]. The increase in the activities of both GPx and peroxidases by the HSL infusion suggests that this treatment modulates the activity of these enzymes. Therefore, it favors an increase in the activity of these enzymes, thus contributing to the decrease of chronic OS in the kidney of the MS rats. This beneficial effect of the infusion may be associated to the effect of resveratrol and polyphenols [55]. Peroxidases may lower the increase in H_2_O_2_ in patients with MS. An inhibition of the activity of peroxidases diminishes endothelial damage and severe proteinuria and may result in damage to the renal function [11].

GST is the enzyme involved in the metabolism of xenobiotics and in the protection against damage caused by peroxidized. It catalyzes the ionization of GSH to a nucleophilic thiolate anion form which reacts spontaneously with nucleophilic components that are located close nearby such as α,β-unsaturated aldehydes. This reaction is followed by the conjugation of GSH. This conjugation increases the solubility of the toxic products, facilitating their excretion from the cell. The GST activity decreases the LPO rate [19]. Our results show that the GST activity was decreased in the kidney of MS rats. The IR and hyperinsulinemia present in MS can contribute to the GST inhibition [19]. In this sense, the GSTA4 expression is down regulated in the adipose tissue from obese insulin- resistant C57BL/6 mice and in humans with obesity and IR [56]. Moreover, a decrease in GST activity has been related to an increase in ROS in arterial hypertension [57]. However, the HSL treatment showed a tendency to increases the GST activity. This suggests that the decreased GST activity in the kidney of the MS rats favors OS.

The damage caused by OS is evidenced by the increase in LPO and carbonylation and by the decrease of the TAC and GSH. The HSL treatment decreased these damages. The antioxidant properties of the HSL on GST activity and on GSH have been attributed to phenolic compounds such as anthocyanin and protocatechuic acid. GSH contributes to the decrease LPO and carbonylation which in turn, increases the GSH and TAC in the kidney. In the aortic aneurysm of Marfan syndrome patients, the infusion of 2% HSL increased GST activity, GSH and TAC concentration but LPO and carbonylation levels were decreased [50]. These effects might be due to polyphenols present in the HSL which have a benzoic ring with one or more hydroxyl groups that participate as scavengers of ROS They also participate in a second line of defense when they have not been neutralized by the enzymatic antioxidant system [55]. This suggests that polyphenols can contribute to increase the antioxidant capacity of the non- enzymatic system, favoring an increase of TAC and GSH and a decrease in LPO and carbonylation levels in the kidney of MS rats. The TAC is considered as a reliable indicator of the antioxidant content and it depends on the enzymatic and non-enzymatic antioxidant system in which GSH participates. In this sense, GSH is the most versatile antioxidant since it has a variety of functions that include the detoxification of xenobiotics or their metabolites. It is the largest source of endogenous antioxidants, and it inhibits the radicals OH^−^, O_2_^−^. It regenerates vitamins C and E, reconverting them to their active forms. It acts as a cofactor for the GPx and GST and as a substrate of GR. It is transported by amino acids through the plasma membrane acting as a storage source of cysteine. Eighty-five percent of the total cellular GSH is free, and the rest is bound to proteins [44]. Our results show that the GSH levels were increased by the HSL treatment. This effect may be due to the increase in the genetic expression of γ-glutamyl-cysteine synthetase and GSH synthetase caused by HSL. This enzyme is responsible for the de novo synthesis of GSH [56].

For its part, GR is the enzyme that catalyzes the reaction by which oxidized glutathione (GSSG) is reduced to GSH using NADPH^+^ as an energy source. GR was the only enzyme that was increased in the kidney of rats with SM. This may be due to the OS that is associated with the decrease in the GSH concentration and that results in a compensatory mechanism that is aimed to increase GSH levels [57,58]. However, the increase of the GSH levels by the HSL treatment decreases the activity this enzyme.

In addition, Vit C contributes to the increase in TAC. This vitamin is water- soluble having a potent antioxidant effect in vivo. Its basal concentration in plasma is within the μM range. However, its concentration is decreased in the presence of OS [59]. Our results show that the concentration of Vit C in the kidney was lower in MS and that the HSL treatment increased its concentration. The calyces of HSL are rich in organic acids such as citric and ascorbic acid [16]. The input of Vit C by the infusion of HSL favors the elevation of the levels of this vitamin and contributes to GSH regeneration, which favors the reduction of LPO and carbonylation.

## 4. Material and Methods

### 4.1. Animals

The experiments with laboratory rats were approved by the Laboratory Animal Care Committee of our institution and were conducted in compliance with the Guide for the Care and Use of Laboratory Animals of NIH. Eight male 250–300 g weight rats were used per each of the following groups: group 1 control, group 2 MS with 30% sucrose in their drinking water for 12 weeks and Group 3 MS with 30% sucrose and an HSL infusion (MS + HSL) at the concentration of 20 g/L (2%). The animals were housed in ad hoc plastic boxes (Nalgene, New York, NY, USA) and were subjected to 12 h light/obscurity cycles and environmental temperature between 18 and 26 °C. The rats were fed commercial rodent pellets (LabDiet 5008, PMI Nutrition International, Inc., Richmond, IN, USA). Rodent commercial food for rodents that contained 23% of crude protein, 4.5% of crude fat, 8% of ashes, and 2.5% of added minerals) ad libitum. At the end of the experimental period, the rats were weighed, and their systolic blood pressure (SBP) was determined using a tail cuff attached to a pneumatic pulse transducer (Narco Bio-Systems Inc., Houston, TX, USA), in compliance with the method described by Pérez-Torres et al. [60].

### 4.2. HSL Infusion

The HSL calyces were acquired in Chilapa de Alvarez (high zone from Guerrero, Mexico). The infusion was prepared as follows: 20 g of the HSL calyces was added to a liter of boiling (95–100 °C) drinking water for 10 min and then left to cool. 300 g sucrose was added. The solution was filtered and stored at 4°C until used [60]. Total estimation of vitamin C in the infusion was determined by the method of Jagota [61]. For this determination, 100 μL of the HSL infusion were added to 200 μL of Folin-Ciocalteu reagent 0.20 mM. The mixture was shaken vigorously in a vortex for 5 s and incubated for 10 min. The absorbance was measured at 760 nm. The calibration curve was obtained using an ascorbic acid standard solution. Total flavonoid content in the infusion was determined by the method of Jia [62]. 100 μL of HSL infusion were added to 2175 μL of distilled H_2_O plus 75 μL of 5% NaNO_2_ and incubated for 3 min. Then, 150 μL of 10% AlCl_3_ were added, and the solution was incubated for 5 min. 0.5 mL of 1 M NaOH were added to the mixture and it was shaken vigorously in vortex. The absorbance was measured at 510 nm. The calibration curve was obtained using quercetin as a standard. To determine total anthocyanin content in the infusion, 100 μL were added to 50 mL of buffers (NaC_2_H_3_O_2_, 4 M) at pH 1 and 4.5, respectively, and the absorbance was measured at 520 and 700 nm and compared against a blank cell, filled with distilled H_2_O. The difference in the absorbance was used for calculating the cyanidin-3-glucoside (total monomeric anthocyanin) as described by the method of Lee [63]. The 2% HSL infusion contained 1.13 ± 0.12 mM of vitamin C, 12.37 ± 0.42 mg/L of quercetin and 101± 49 mg/L of cyanidin-3-glucoside.

### 4.3. Albuminuria and Creatinine Depuration

Albuminuria was measured using the bromocresol green reagent [64]. Urine and serum creatinine (UCr and SCr respectively) was measured by the Jaffe method [65] and glomerular filtration was calculated according to the following formula: clearance creatinine (CCr) = [UCr]/[SCr] × urinary volume (24 h)/time (1440 min).

### 4.4. Isolated Perfused Kidney

The right kidney was exposed by a midline laparotomy, and the mesenteric and right renal arteries were cleared of surrounding tissue. The right renal artery was cannulated through the mesenteric artery to avoid interruption of blood flow; and the kidney was removed, suspended, and perfused at constant flow by means of a peristaltic pump (MasterFlex Easy-load II, number 77,200–50; Cole-Parmer Instrument Co, Vernon Hills, IL, USA) with Krebs solution at 37 °C and oxygenated with 95% O_2_/5% CO_2_. The solution had the following composition (mM/L): 118 NaCl, 1.2 NaH_2_PO_4_, 25 NaHCO_3_, 4.7 KCl, 1.2 CaCl_2_, 4.2 MgSO_4_, and 5.5 glucose (pH 7.4). Flow was adjusted to a basal perfusion pressure (PP) of 75 to 90 mmHg. Mean flow rate of the perfusing solution was 8 to 9 mL/min. PP was measured with a transducer (Grass Telefactor, Grass Technologies, Astro Med, West Warwick, RI, USA), captured, and recorded by means of a Grass model polygraph 79D and an online program (Grass PolyView Data Acquisition and analysis version 2.0), in compliance with the method described by Pérez-Torres et al. [66]. After a period of 20 min of equilibrium, we proceeded to administer boluses of vasoconstrictors and vasodilators, which were given for sufficiently long intervals to allow the PP to return to baseline (between 75 and 90 mmHg). The sequences and concentrations of the boluses were: norepinephrine (NE) 20μM, NE 20μM plus acetylcholine (Ach) 20μM, and finally NE 20μM plus 1.7 μM of H_2_O_2_.

Changes in the PP produced by the NE, NE+Ach and NE+H_2_O_2_ were calculated by taking the mean of the pulsatile traces before the administration and the mean of the traces at the maximal PP value after administration. Data are expressed as changes delta (Δ) of PP in mmHg. After each perfusion bolus, the kidneys were washed for a period of 20 min with Krebs solution, to allow it to return to the basal PP (75–90 mmHg), and no sign of tachyphylaxis was present.

### 4.5. Serum Sample

The abdominal aorta was exposed by midline laparotomy and cannulated to obtain 4 mL of blood, taking care to avoid hemolysis. The blood was centrifuged for 20 min at 936 g and at 4 °C, in order to collect the serum in aliquots of 400 mL and stored at −30 °C.

### 4.6. Biochemical Variables

Commercial kits were used for the determination of some serum biochemical variables in the rats. Glucose concentration was determined by enzymatic SERA-PAKR Plus kit (Bayer Corporation, S’ees, France). TC and TG determinations were made using commercial enzymatic kits, (RANDOX Laboratories Ltd., Crumlin, County Antrim, UK). Leptin and insulin were determined using commercial radioimmunoassay kits (RIA) (Linco Research Inc., Saint Charles, MO, USA). The HOMA index of resistance to insulin was calculated. HOMA−IR = insulin μU/mL × glucose mM/L/22 5.

### 4.7. Kidney Homogenate

The left kidney was dissected and washed with 0.9% saline solution and immediately perfused with a sucrose buffer (25 mM sucrose, 10 mM Tris, 1 mM EDTA, and pH 7.35). The capsule was removed, and the kidney was homogenized in the same sucrose buffer with protease inhibitors (1 mM PMSF, 2 μM pepstatin, 2 μM leupeptin, and 0.1% aprotinine). The homogenate was kept in ice. The kidney homogenate was centrifuged at 900 g for 10 min at 4 °C. The supernatant was separated and stored at −30 °C until required. Total proteins were determined by the Bradford method [67].

### 4.8. Lipid Peroxidation

Fifty μL of CH_3_OH with 4% BHT plus phosphate buffer pH 7.4 were added to 100 μg of kidney homogenate. The mixture was shaken vigorously in a vortex for 5 s and then incubated in a water bath at 37 °C for 30 min. 1.5 mL of 0.8 M thiobarbituric acid were then added and the sample was incubated in a water bath at boiling temperature for 1 h. After this time and to stop the reaction, the samples were placed on ice; 1 mL 5% KCl was added to each sample as well as 4 mL n-butanol. Samples were shaken in a vortex for 30 s and centrifuged at 4000 rpm at room temperature for 2 min. Then the n-butanol phase was extracted, and the absorbance was measured at 532 nm [68].

### 4.9. Evaluation of Total Antioxidant Capacity

One hundred μg of kidney homogenate ware suspended in 1.5 mL of a reaction mixture prepared as followed: 300 mM acetate buffer pH 3.6, 20 mM hexahydrate of ferric chloride, 10 mM of 2,4,6-tris-2-pyridil-s-triazine dissolved in 40 mM chlorhydric acid. The solutions were added in a relation of 10:1:1 *v*/*v*, respectively. The mixture was shaken vigorously in a vortex for 5 s. It was then incubated at 37 °C for 15 min in the dark. The absorbance was measured at 593 nm [59].

### 4.10. Vitamin C

One hundred μg of kidney homogenate were added to 20% trichloroacetic acid. After vigorous shaking the samples were kept in an ice bath for 5 min and centrifuged at 5000 rpm for 5 min. 200 μL of Folin-Ciocalteu reagent 0.20 mM were added to the supernatant. The mixture was shaken vigorously in a vortex for 5 s and incubated for 10 min. The absorbance was measured at 760 nm [59].

### 4.11. Carbonylation

One hundred μg of kidney homogenate were added to 500 μL of HCl 2.5 N. Another sample with 500 μL of 2,4-dinitrophenylhydrazine (DNPH) and incubated in the dark at room temperature for one hour, shaking with a vortex every 15 min was run in parallel. At the end of the incubation period, 500 μL of 20% trichloroacetic acid were added, and the sample was centrifuged at 15,000× *g* for 5 min. The supernatant was discarded. Two washings were performed, first removing the precipitate with a sealed capillary tube by adding 1 mL ethanol/ethyl acetate. It was incubated for 10 min, and centrifuging at 15,000× *g* for 10 min. Finally, 1 mL of 6 M guanidine hydrochloride in 20 mM KH_2_PO_4_ pH 2.3 was added. The mixture was incubated again at 37 °C for 30 min. Absorbance was read in a spectrophotometer at 370 nm, using water bi-distilled as blank and a molar absorption coefficient of 22,000 M^−1^ cm^−1^ [59].

### 4.12. GSH Concentration

One hundred μg of kidney homogenate previously deproteinized with 20% trichloroacetic acid (vol/vol) and centrifugated to 10,000× *g* for 5 min, were added to 800 μL of phosphate buffer 50 mM, pH 7.3, plus 100 μL of Ellman’s reagent (5, 5′-dithiobis-2-nitrobenzoic acid) 1 M. The mixture was incubated at room temperature for 5 min and absorbance was read at 412 nm [59].

### 4.13. NO_3_^−^/NO_2_^−^ Ratio

The NO_3_^−^ was reduced to NO_2_^−^ by the nitrate reductase enzyme reaction. 100 μL of plasma previously deproteinized with 0.5 N, NaOH and 10%, ZnSO_4_ were mixed, and the supernatant was incubated for 30 min at 37 °C in presence of nitrate reductase (5 units). At the end of the incubation period, 200 µL of sulfanilamide 1% and 200 µL of N-naphthyl-ethyldiamine 0.1% were added and the total volume was adjusted to 1 mL. The absorbance was measured at 540 nm [59].

### 4.14. Superoxide Dismutase, Peroxidase and Catalase Activities in Native Gels

One hundred µg of kidney homogenate were used for measurement of the activity of the SOD isoforms, peroxidase, and CAT. The determinations were performed in 10% polyacrylamide native gels. SOD and CAT isoforms were revealed according to the methods that were previously described by Pérez-Torres et al. [28]. Purified SOD from bovine erythrocytes with a specific activity of 112 U/mg of protein (Sigma-Aldrich, St. Louis, MO, USA) and purified CAT from a bovine liver having a specific activity of 60 U/mg (Sigma-Aldrich) were used as positive controls. The previously mentioned antioxidant enzyme activity determinations were performed according to the manufacturer’s instructions. Samples were placed in a separate lane of the gel and run in parallel with the biological samples. The intensity of the signal from the controls was used as a reference to measure the enzymatic activity in the tissue samples. Therefore, the results are expressed as U of activity per mg of protein. The gels were analyzed by densitometry with the Kodak Image^®^ 3.5 [68].

For the peroxidase activity, 35 μL of horseradish peroxidase was loaded to a final concentration of 178.5 μg as a standard and 100 μg of protein in the same conditions of the native gel were run as previously described. To observe the activity of the peroxidases, the gel was washed with distilled water three times, during 5 min, and it was then incubated with a mixture of 0.003 mg/mL 3,3,5,5-tetramethylbenzidine dissolved in a solution of ethanol: acetic acid: water (1:1:1) with H_2_O_2_ for 10 min in the dark. In these conditions, where peroxidases are present, the gel remains transparent and 3,3,5,5-tetramethylbenzidine is oxidized showing a green coloration. The gels for peroxidase activities were analyzed by densitometry with a Kodak Image^®^ 3.5 system, and activities were calculated following the technique described above for SOD isoforms and CAT [19].

### 4.15. Enzymes That Use Glutathione and/or Oxidized Glutathione

For the GPx, GST, and GR activities, 100 μg of kidney homogenate were utilized according to previously described methods [56]. The GPx activity is expressed as nmol of NADPH oxidized/min/mg protein, with an extinction coefficient of 6220 M^−1^ cm^−1^ at 340 nm for NADPH. The GST activity is expressed as units of GS-TNB mol/min/mg protein with an extinction coefficient of 14,150 M^−1^ cm^−1^. The GR activity is expressed as μmol of reduced GSSG/min/mg protein, with an extinction coefficient of 6220 M^−1^ cm^−1^.

### 4.16. Histopathological Analysis

Tissue was processed for light microscopy according to standard techniques. A segment of kidney was dissected, decapsulated and washed in 0.9% NaCl for 30 s, fixed in 10% formalin solution for 24 h, gradually dehydrated in ethanol, cleared in xylene, and embedded in paraffin. The kidney was cut into five-micrometer-thick slices with a microtome (Leica RM212RT, Wetzlar, Germany); the paraffin sections were stained with periodic acid–Schiff stain (PAS). Histological sections were analyzed at 400× magnification using a model 63300 light microscope (Carl Zeiss, Oberkochen, Germany), equipped with a Tucsen (9 megapixels) digital camera equipped with the TSview 7.3.1 software. The glomerular area was analyzed by densitometry using the Sigma Scan Pro 5 Image Analysis software, (Systat Software Inc., San Jose, CA, USA). The density values are expressed as pixel units.

### 4.17. Statistical Analysis

The Sigma Plot 14 program (Systat Software Inc. 2107, San Jose, CA95131 EE.UU. North First Street, Suite 360) was used to generate the graphs and to perform the statistical analyses. The data are presented as the mean ± SE. Statistical significance was determined by one-way ANOVA test, followed by Tukey’s post hoc test. Differences were considered as statistically significant when *p* ≤ 0.05.

## 5. Conclusions

**Figure 7:**
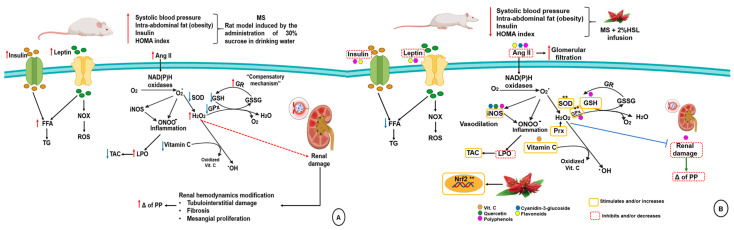
Panel (**A**) renal damage in metabolic syndrome on the alteration of the enzymatic and non-enzymatic antioxidant systems. Panel (**B**) Effect of 2% HSL infusion on enzymatic and non-enzymatic antioxidant systems in kidney of the MS rats. Abbreviations: **FFA** = free fatty acids, **H_2_O_2_** = hydrogen peroxide, **HSL** = *Hibiscus sabdariffa* L., **GPx** =glutathione peroxidase, **GR** = glutathione reductase, **GSH** = glutathione, **GSSG** = oxidized glutathione, **LPO** = lipoperoxidation, **MS** = metabolic syndrome **NOX** = nicotinamide adenine dinucleotide phosphate oxidase, i**NOS** = inducible nitric oxide, **O_2_^−^** = superoxide anion, **OH** = hydroxyl radical, **ONOO^−^** = peroxynitrate, **Prx** = peroxidases, **ROS** = reactive oxygen species, **SOD** = superoxide dismutase, **TAC** = total antioxidant capacity, **TG** = triglycerides.

Treatment with a 2% HSL infusion protects renal function in a MS rat model through the natural antioxidants present in it. These compounds contribute to decrease body weight, TG, insulin, HOMA index and leptin levels. Furthermore, they favor the correct renal vascular response, which contributes to the increase in the glomerular filtration rate leading to a better CCr, which reduces hypertension. In addition, the HSL infusion also promotes an increase in the enzymatic and non-enzymatic antioxidant systems leading to a decrease in OS and reversing the impaired renal function. Figure 7 summarizes the beneficial effect of 2% HSL infusion in the protection of renal damage in a rat model of MS.

## Figures and Tables

**Table 1:** General characteristic of experimental groups.

Variables	Control	MS	MS + HSL
Glucose (mmol/L)	114.7 ± 6.8	113.5 ± 5.0	118.5 ± 4.9
Insulin (μU/mL)	5.3 ± 0.5	17.6 ± 1.2 **	11.6 ± 1.1 *
HOMA index	2.0 ± 0.1	9.9 ± 1.8 **	4.2 ± 0.3 *
Triglycerides (mg/dL)	52.5 ± 4.6	139.5 ± 25.6 **	74.1 ± 5.9 *
Cholesterol (mg/dL)	46.1 ± 2.1	51.5 ± 1.7	51.2 ± 0.8
Intra-abdominal fat (g)	3.1 ± 0.3	11.6 ± 1.3 **	6.1 ± 0.4 *
Systolic blood pressure (mmHg)	119.1 ± 4.9	140.1 ± 6.9 **	132.2 ± 4.4 *
Leptin (ng/mL)	1.9 ± 0.5	13.5 ± 7.8 **	3.6 ± 0.8 *
Body mass (g)	397.8 ± 26.6	556.8 ± 14.1 *	425.0 ± 27.6 *

Data are mean ± SE, *n* = 8 each group. Statistically significant at * *p* < 0 04 control and MS + HSL vs. MS; ** *p* = 0 001 control vs. MS. Abbreviations: MS = metabolic syndrome; MS + HSL = metabolic syndrome plus *Hibiscus sabdariffa* L.

**Table 2:** Renal function indicators in the experimental groups.

Variables	Control	MS	MS + HSL
Drinking wáter (mL/24 h)	18.12 ± 2.30	58.75 ± 5.06 **	35.00 ± 5.08 ^§^
Weight of the right kidney (g)	1.27 ± 0.03	1.51 ± 0.08 ^†^	1.30 ± 0.04 *
Urine (mL/24 h)	13.62 ± 2.53	24.00 ± 4.20 *	27.00 ± 3.33
UCr (mg/dL)	12.32 ± 1.29	3.95 ± 0.36 **	6.76 ± 1.19
SCr (mg/dL)	0.41 ± 0.02	0.67 ± 0.03 **	0.40 ± 0.02 ^§^
CCr (mL/min)	2.51 ± 0.35	1.45 ± 0.15 **	2.79 ± 0.55 *
Albuminuria (mg/mL)	32.28 ± 5.54	74.67 ± 8.57 **	43.08 ± 7.54 ^§^

Data are mean ± SE, *n* = 8 each group. Statistically significant at * *p* ≤ 0.04 MS + HSL and control vs. MS, ^†^ *p* = 0.01 control vs. MS, ** *p* ≤ 0.003, control vs. MS and ^§^ *p* ≤ 0.001 MS + HSL vs. MS. Abbreviations: MS = metabolic syndrome; MS + HSL = metabolic syndrome plus *Hibiscus sabdariffa* L.

**Table 3:** Oxidative stress indicators in the kidney homogenate in the experimental groups.

Variables (mg of Protein)	Control	MS	MS + HSL
LPO (MDA nmol)	0.34 ± 0.05	0.56 ± 0.03 ***	0.45 ± 0.05 ^††^
Carbonylation (ng carbonyls)	0.07 ± 0.00	0.11 ± 0.01 ***	0.08 ± 0.01
NO_3_^−^ and NO_2_^−^ (nM)	0.09 ± 0.00	0.06 ± 0.01 ^†^	0.10 ± 0.00 ^§^
GSH (μmol)	0.0014 ± 0.0001	0.0009 ± 0.0001 ^†^	0.0013 ± 0.0000 **
TAC (Trolox nmol)	0.61 ± 0.01	0.57 ± 0.01 ^†^	0.60 ± 0.01
Vit C (μM)	0.021 ± 0.002	0.015 ± 0.001 *	0.018 ± 0.000 *

Data are mean ± SE, *n* = 8 each group. Statistically significant at *** *p* ≤ 0.001 control vs. MS, ^†^ *p* ≤ 0.03 control vs. MS, ^§^ *p* ≤ 0.001, MS + HSL vs. MS. ** *p* = 0.01 MS + HSL vs. MS, * *p* = 0.05 control MS + HSL vs. MS, ^††^ *p* = 0.01 MS + HSL vs. MS. Abbreviations: MS = metabolic syndrome; MS + HSL = metabolic syndrome plus *Hibiscus sabdariffa* L., Statistical significance was determined by one-way ANOVA test, followed by Tukey’s post hoc test.

## Data Availability

The datasets generated and analyzed during the current study are available from the corresponding author on reasonable request.

## Sample Availability

Samples of the compounds are available from the authors.
